# Workpiece temperature field and process force data for turning steel and aluminum alloy under varying machining parameters

**DOI:** 10.1016/j.dib.2026.113240

**Published:** 2026-09-04

**Authors:** Lars Langenhorst, Jens Sölter

**Affiliations:** a University of Bremen, MAPEX Center for Materials and Processes, Bibliothekstraße 1, 28359 Bremen, Germany; b Leibniz Institute for Materials Engineering IWT, Badgasteiner Straße 3, 28359 Bremen, Germany

**Keywords:** Cutting, Heat partition, In-process measurement, Manufacturing

## Abstract

Cylindrical workpieces made of AISI 4140 steel and A97075 aluminum alloy were machined in an external longitudinal turning process using uncoated cemented carbide cutting tools without metal working fluid. During machining, the process forces were measured by means of piezoelectric sensors within a dynamometer. In addition, temperature on the workpiece surface was measured using infrared thermography. The dataset provides the process forces as raw data over the process time, along with the calculated mean values and standard deviations for each test point within the investigated design of experiments, in which the depth of cut, spindle rotation speed, and feed rate are varied. The process temperatures measured along a focused line in feed direction are also provided as raw data and mean values over time and corresponding uncertainty for all experiments. In metal cutting, an understanding of the process forces, which act as external loads on the workpiece, and the workpiece temperature, as an internal material load, is closely linked to the subsequent functional performance of the final component. Reuse potential thus lies primarily in explaining modifications of material properties in the surface layer of the workpiece and its geometric deviations due to cutting and also in using the data for validation of modelling approaches. For analyzing tool wear, process forces and temperatures are also important factors. This dataset enables such analyses without having to repeat the time-consuming calibration of temperature measurement systems and preparation of machining tests.

Specifications Table

**Table:** 

Subject	Engineering & Materials science
Specific subject area	Manufacturing Technology involves researching and optimizing machining processes in a material- and function-oriented manner to promote technological progress
Type of data	Table (.xlsx format) Raw, Processed
Data collection	Data was collected during external longitudinal turning cylindrical workpieces made of AISI 4140 steel and A97075 aluminum alloy on a machine tool, type DMC 65 V from Deckel Maho, using uncoated cemented carbide cutting tools, type TPGN160302–883 from Seco Tools. Process forces were measured by a piezoelectric multicomponent dynamometer, type 9257B from Kistler Group, three charge amplifiers, types 5011, and a data acquisition module, type cDAQ-9174 from National Instruments with an Analog-to-Digital Converter, type 9215. Temperature fields of the workpiece surface were measured by an infrared thermography camera, type ImageIR 8300 from InfraTec, with a microscope lens, type M = 1,0x WD 200.
Data source location	Institution: University of Bremen City: Bremen Country: Germany
Data accessibility	Repository name: Zenodo [1] Data identification number: 10.5281/zenodo.21708426 Direct URL to data: https://zenodo.org/records/21708426
Related research article	none

## 1 Value of the Data

• These data are valuable for the manufacturing technology community in terms of understanding the resulting external (forces) and internal material loads (temperatures) acting on the workpiece during the turning process when machining parameters (depth of cut, spindle rotation speed, and feed rate) are systematically varied.
• Researchers and practitioners may benefit from this dataset through its comprehensive compilation including the time-consuming calibration of temperature measurement systems and preparation of machining tests. The data can be reused in order to explain modifications of material properties in the surface layer of the workpiece and its geometric deviations due to cutting as well as analyzing tool wear.
• Mechanisms during cutting are investigated by means of modelling approaches, e.g. numerical chip formation simulations. As these models include complex assumptions, e.g. on modelling friction and plasticity phenomena, validation by comparing calculated with measured results is essential. With measured process forces and workpiece temperatures, the dataset provides the most important quantities for such comparisons. Providing temperature fields instead of discrete single-point values significantly improves the spatial validation quality, allowing models to be benchmarked against realistic thermal gradients and heat-affected zones. Additionally, the systematic variation of the machining parameters provides an ideal baseline to validate the model sensitivity and predictive accuracy across changing conditions. By applying identical machining parameters to two different workpiece materials, the dataset further isolates the influence of thermophysical material behavior on forces and temperature fields, allowing modelers to critically evaluate how accurately their simulations capture variations in temperature dependent material plasticity and thermal properties.
• The acquisition of process forces and temperature fields enables insights into the energy conversion during cutting. Furthermore, the comprehensive documentation of both the experimental conditions and measurement uncertainties strengthen the reliability and physical significance of these experimental findings.

## 2 Background

The primary objective for creating this dataset was to better understand the dissipation of heat in cutting processes. It is well known, that nearly all of the supplied mechanical energy due to the kinematics in cutting (corresponding to the product of cutting force and cutting velocity) is converted into heat due to shearing and friction. As heat almost always promotes mechanisms unfavorable for the final workpiece state, e.g. generation of brittle layers or residual tensile stresses, understanding and minimizing the fraction of heat into the workpiece is a common goal in cutting [2]. Besides heat flow into the workpiece, relevant amount of heat can also flow into the chip and the cutting tool, making the heat dissipation a complex interaction phenomenon of solid-state convection due to material flow and conduction. In order to investigate this phenomenon for complex kinematics in cutting processes, analytical and numerical thermal models have been developed and partly validated for milling processes [3,4]. Attempts to validate these models for turning have so far been hindered by a lack of experimental data, which has been the starting point for creating the dataset.

## 3 Data Description

**Fig. 1:**
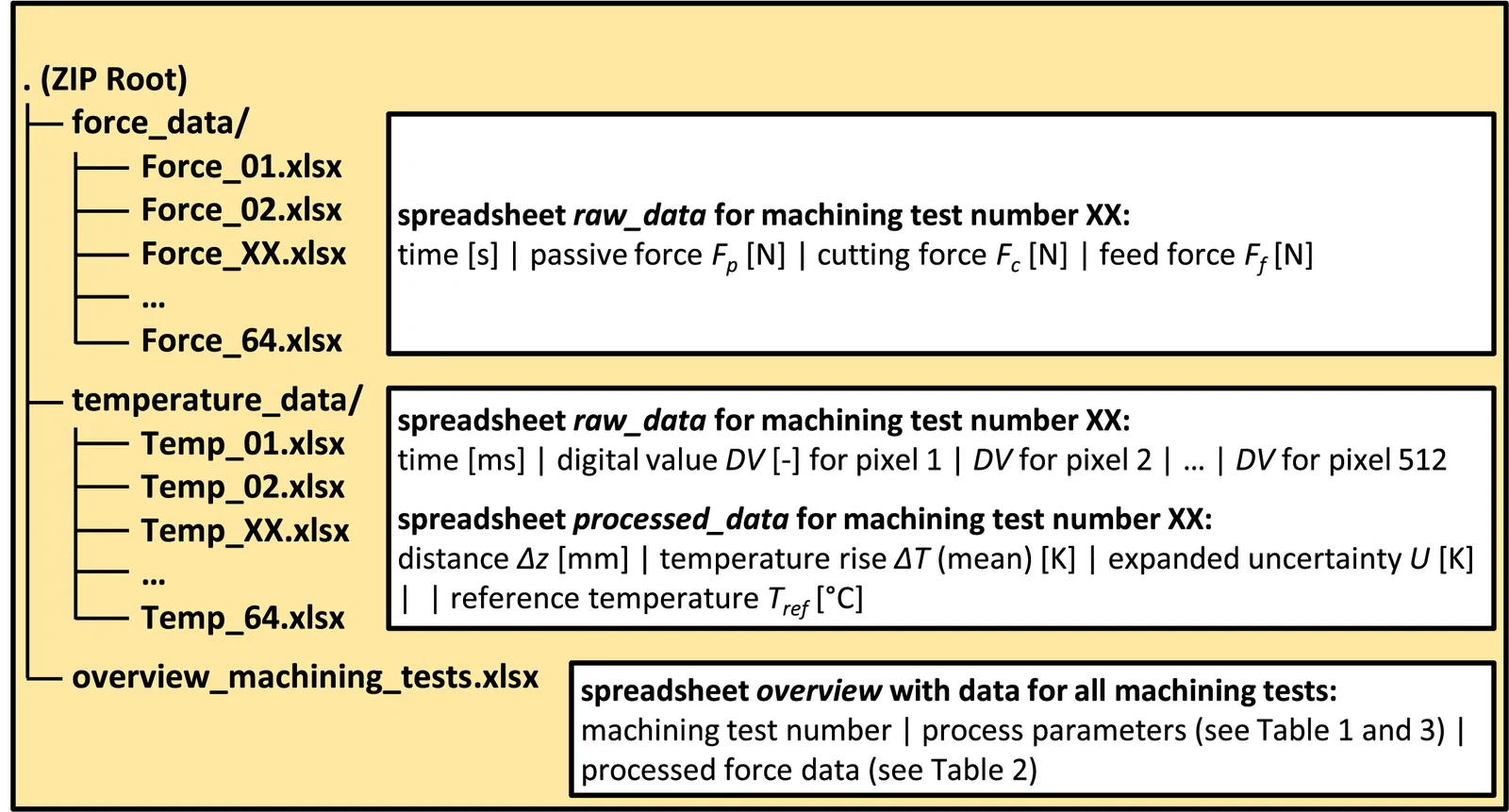
Repository folder structure and content of folders and files including file-naming convention.

The dataset in the repository is provided as one file in .zip format, see Fig. 1.

In the root directory of the ZIP archive, the file *overview_machining_tests.xlsx* contains details on the 64 conducted machining tests, according to Table 1 and Table 3, and processed data of the force measurements, according to Table 2.

**Table 1:** Columns A to F in file overview_machining_tests.xlsx, exemplary for machining test no 31.

machining test number	workpiece material	tool cutting edge angle *κ* [°]	spindle rotation speed *n* [1/min]	feed rate *v_f_* [mm/min]	depth of cut *a_p_* [mm]
31	A97075	91	2387.32	650	0.5

**Table 2:** Columns G to L in file overview_machining_tests.xlsx, exemplary for machining test no 31.

cutting force *F_c_* (mean) [N]	feed force *F_f_* (mean) [N]	passive force *F_p_* (mean) [N]	cutting force *F_c_* (standard deviation) [N]	feed force *F_f_* (standard deviation) [N]	passive force *F_p_* (standard deviation) [N]
147.29	36.8	51.38	3.29	1.76	1.64

**Fig. 2:**
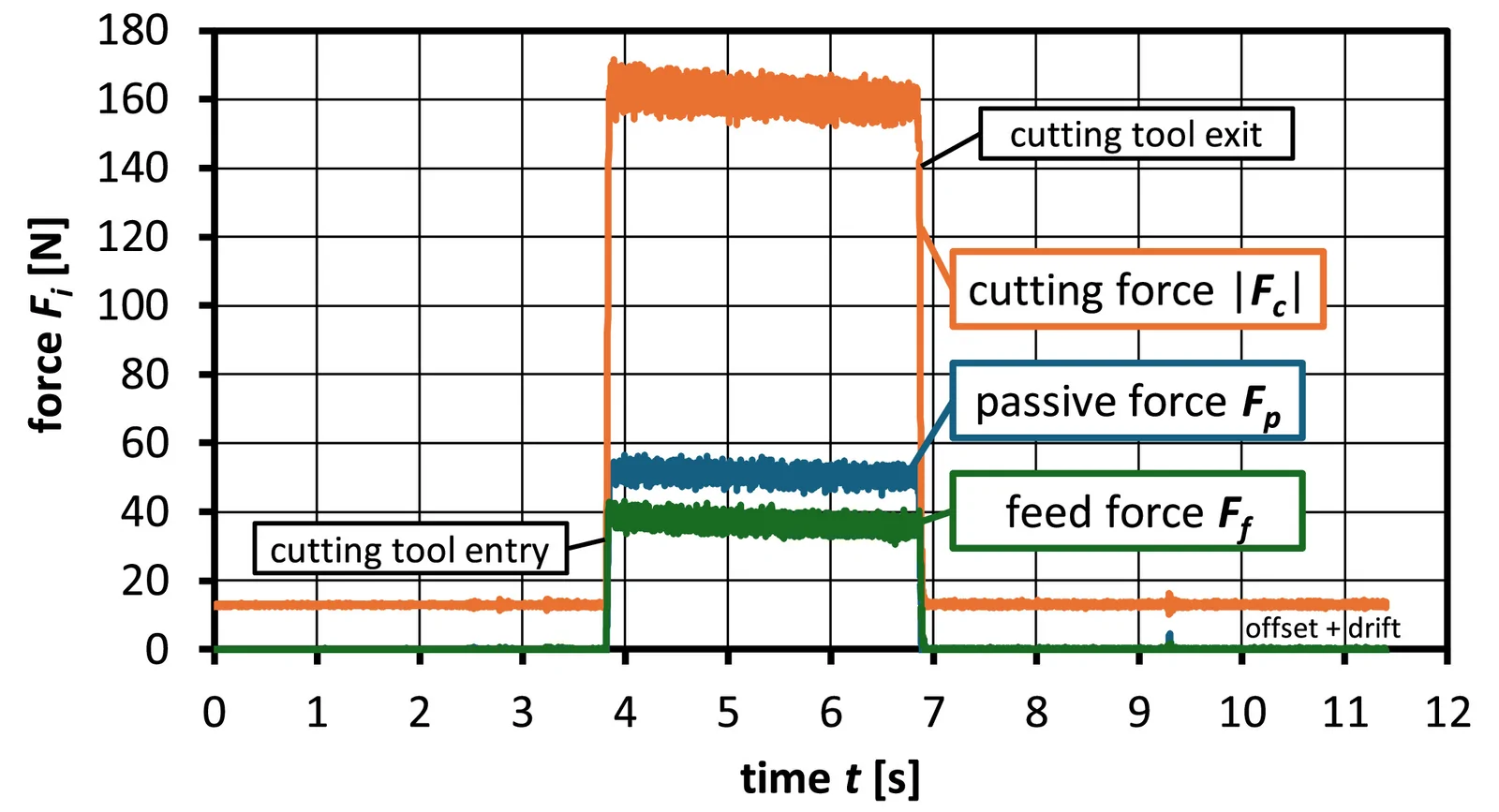
Raw data of the force measurement for machining test no 31.

Also in the root directory, the folder *force_data* contains the raw data of the force measurements, provided in one file per machining test (column A: time, column B: passive force, column C: cutting force, column D: feed force) and indicating the machining test number in the file name, e.g. *Force_31.xlsx*. Fig. 2 shows the three force signals for machining test number 31.

**Fig. 3:**
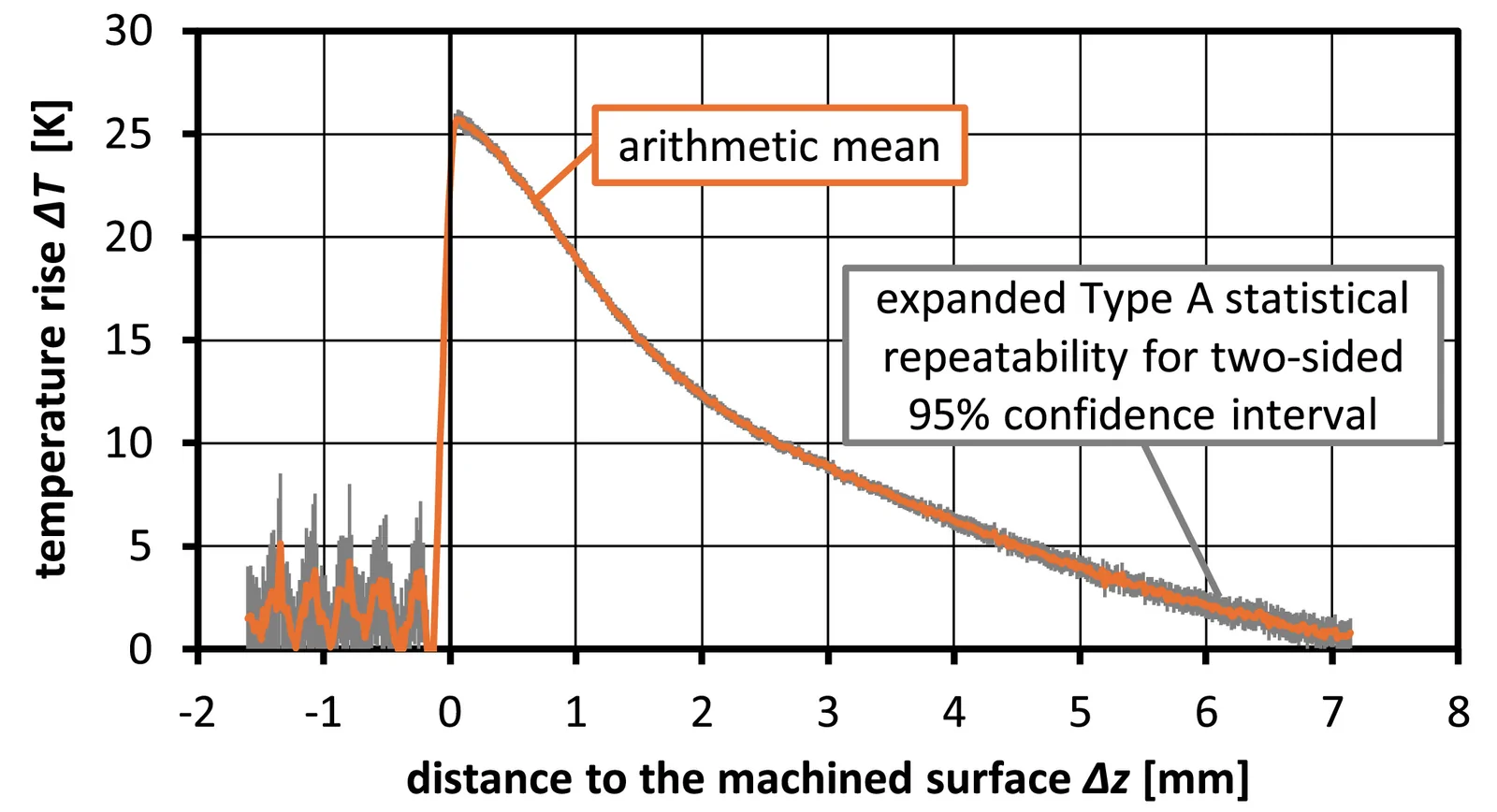
Processed data of the temperature measurement for machining test no 31.

In the second folder *temperature_data* within the root directory, data from temperature measurements are provided, again in one file per machining test, e.g. *Temp_31.xlsx*. In the first worksheet *raw_data*, the digital value signal *DV* from the infrared camera is given pixelwise along the line L1 in focus of the camera, see Fig. 5, for each measured time step (column A: time). Herein Columns B through SS store the signal values for pixels 1 to 512 (indexed from bottom to top). In the second worksheet *processed_data*, according to the description of the data preparation in the following section on temperature field measurement, column A contains the distance to the machined surface *Δz*, column B the process-induced temperature rise *ΔT* (mean), column C the expanded uncertainty *U*, and cell E2 the reference temperature *T_ref_*. Fig. 3 shows this processed data for the machining test number 31, corresponding to Fig. 5. For metrological clarity, it is important to note that the expanded uncertainty refers strictly to the expanded Type A statistical repeatability to illustrate local temporal precision. It does not represent the complete expanded measurement uncertainty, as it excludes the calibration uncertainty described in the following section on temperature field measurement.

Table 3 shows the complete experimental matrix with details on the 64 conducted machining tests, including repetition tests with the same parameters.

**Table 3:** Complete experimental matrix (columns A to F in file overview_machining_tests.xlsx).

machining test number	workpiece material	tool cutting edge angle *κ* [°]	spindle rotation speed *n* [1/min]	feed rate *v_f_* [mm/min]	depth of cut *a_p_* [mm]
01	AISI 4140	91	2387.32	250	0.5
02	AISI 4140	91	2387.32	250	1.0
03	AISI 4140	91	2387.32	250	1.5
04	AISI 4140	91	2387.32	450	0.5
05	AISI 4140	91	2387.32	450	1.0
06	AISI 4140	91	2387.32	450	1.5
07	AISI 4140	91	2387.32	650	0.5
08	AISI 4140	91	2387.32	650	1.0
09	AISI 4140	91	2387.32	650	1.5
10	AISI 4140	91	3978.87	250	1.0
11	AISI 4140	91	3978.87	250	1.5
12	AISI 4140	91	3978.87	450	0.5
13	AISI 4140	91	3978.87	450	1.0
14	AISI 4140	91	3978.87	450	1.5
15	AISI 4140	91	3978.87	650	0.5
16	AISI 4140	91	3978.87	650	1.0
17	AISI 4140	91	5570.42	250	1.0
18	AISI 4140	91	5570.42	250	1.5
19	AISI 4140	91	5570.42	450	0.5
20	AISI 4140	91	5570.42	450	1.0
21	AISI 4140	91	5570.42	450	1.5
22	AISI 4140	91	5570.42	650	0.5
23	AISI 4140	91	5570.42	650	1.0
24	AISI 4140	91	5570.42	650	1.5
25	AISI 4140	91	3978.87	450	1.0
26	A97075	91	2387.32	250	1.0
27	A97075	91	2387.32	250	1.5
28	A97075	91	2387.32	450	0.5
29	A97075	91	2387.32	450	1.0
30	A97075	91	2387.32	450	1.5
31	A97075	91	2387.32	650	0.5
32	A97075	91	2387.32	650	1.0
33	A97075	91	2387.32	650	1.5
34	A97075	91	3978.87	250	0.5
35	A97075	91	3978.87	250	1.0
36	A97075	91	3978.87	250	1.5
37	A97075	91	3978.87	450	1.0
38	A97075	91	3978.87	450	1.5
39	A97075	91	3978.87	650	0.5
40	A97075	91	3978.87	650	1.5
41	A97075	91	5570.42	250	0.5
42	A97075	91	5570.42	250	1.0
43	A97075	91	5570.42	250	1.5
44	A97075	91	5570.42	450	0.5
45	A97075	91	5570.42	450	1.0
46	A97075	91	5570.42	450	1.5
47	A97075	91	5570.42	650	0.5
48	A97075	91	5570.42	650	1.0
49	A97075	91	5570.42	650	1.5
50	A97075	91	3978.87	450	1.0
51	AISI 4140	91	2387.32	250	4.0
52	AISI 4140	91	5570.42	250	4.0
53	A97075	91	2387.32	250	4.0
54	A97075	91	5570.42	250	4.0
55	AISI 4140	45	3978.87	250	1.0
56	AISI 4140	45	3978.87	450	1.0
57	AISI 4140	45	3978.87	650	1.0
58	AISI 4140	45	3978.87	250	1.0
59	A97075	45	3978.87	450	1.0
60	A97075	45	3978.87	650	1.0
61	A97075	45	3978.87	250	1.0
62	A97075	45	3978.87	450	1.0
63	AISI 4140	91	3978.87	450	1.5
64	A97075	91	3978.87	450	0.5

## 4 Experimental Design, Materials and Methods

### 4.1 Experimental setup

Turning experiments were conducted on a machine tool, type DMC 65 V from Deckel Maho, with specifics detailed in Table 4.

**Table 4:** Technical specifications of the machine tool Deckel Maho DMC 65V.

CNC Controller	Heidenhain TNC 430
**workspace (X / Y / Z)**	650 / 500 / 500 mm
**positioning accuracy**	± 0.005 mm
**repeatability**	± 0.002 mm
**max. spindle speed**	18,000 rpm
**max. spindle power**	15 kW
**max. spindle torque**	130 Nm

**Fig. 4:**
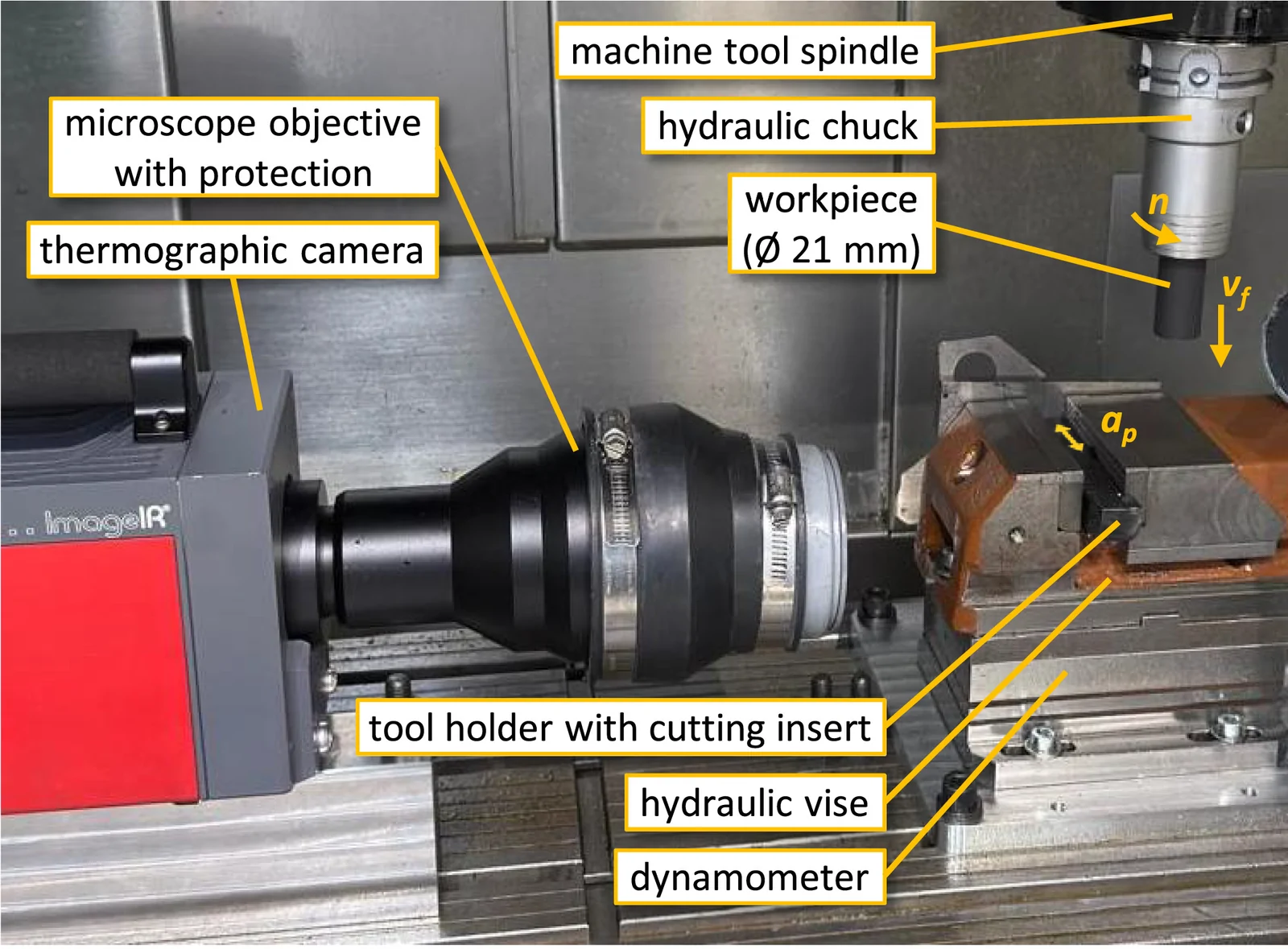
Experimental setup.

Fig. 4 shows the corresponding setup in the machine tool. The machine tool spindle is equipped with a hydraulic chuck, type 16.510.63.20.Z/100 from WTE, in order to clamp the workpiece with low concentricity deviation during rotation with spindle rotation speed *n*. The workpiece material was purchased from Alois Schmitt GmbH & Co. KG as (a) steel AISI 4140 round bar (bright rolled, quenched and tempered, peeled) with a diameter of 22 mm according to EN10277 and (b) aluminum alloy A97075 round bar (extruded, solution heat-treated and aged according to T6) with a diameter of 25 mm according to EN 573–3 and EN 755–1/2/3. In sample preparation, the round bars were sawn to a length of 90 mm and turned to a diameter of 21 mm, with a clamping journal of 20 mm in diameter over a length of 50 mm. Prior to turning, the steel samples were heat-treated to a ferritic-pearlitic state (heating at 10 K/min to 850 °C, holding at 850 °C for a duration of 120 min, oven cooling at 1.7 K/min). Following sample preparation, the workpieces exhibited a surface hardness of (a) 211 HV1 and (b) 185 HV1. For the thermographic measurements, the workpieces were coated with an oven paint from Ulfalux, color pearl-matte black. Uncoated cemented carbide cutting inserts, type TPGN160302–883 from Seco Tools, were used. These were screwed onto a tool holder with either (a) a 91° tool cutting edge angle of type CTGPL2020–16 from Seco Tools, or (b) a 45° tool cutting edge angle of type CTDPL2525–16 from Sandvik. The tool-side setup was aligned such that the main cutting edge of the insert is in radial alignment with the workpiece center at an inclination angle of 0°. Details on the cutting tool geometries are summarized in Table 5.

**Table 5:** Cutting tool geometries depending on the tool holder.

tool holder	tool cutting edge angle κ [°]	rake angle *γ* [°]	clearance angle *α* [°]	nose radius *r_ε_* [mm]	cutting edge radius *r_β_* [µm]
CTGPL2020–16, Seco	91	5	11	0.2	5.4
CTDPL2525–16, Sandvik	45	6			

The tool holder is clamped in a hydraulic vise from Allmatic, and its radial position can be adjusted using spacer plates of defined thickness to adapt the depth of cut *a_p_* for the machining process. The vise is fixed to a dynamometer and the machine table. Details regarding the measurement techniques are explained in the following subchapters.

### 4.2 Force measurement

Forces were measured by a piezoelectric multicomponent dynamometer, type 9257B from Kistler Group in all three cartesian axes, corresponding to the cutting, feed, and passive force. Signal processing was performed via three charge amplifiers, type 5011 with a low-pass filter of 300 Hz, and a data acquisition module, type cDAQ-9174 from National Instruments with an Analog-to-Digital Converter, type 9215 and a USB interface to the measurement computer, on which the three signals were recorded at a sample rate of 1 kHz and subsequently saved in .xlsx format. For the initial calibration of the measurement chain, a defined static force of 100 N was subsequently applied in all three spatial directions. Processing of the raw data was done by (1) trimming the signal from tool entry (first point in time reaching a force gradient of at least 1 N/ms) to tool exit (first point in time reaching a force gradient of at least −1 N/ms), (2) applying a zero offset-correction of the signal based on the tool entry force value and a linear drift-correction based on the tool exit force value, (3) calculating the absolute value of arithmetic mean and the standard deviation within the complete interval between tool entry and exit, and (4) save both values in .xlsx format.

### 4.3 Temperature field measurement

**Fig. 5:**
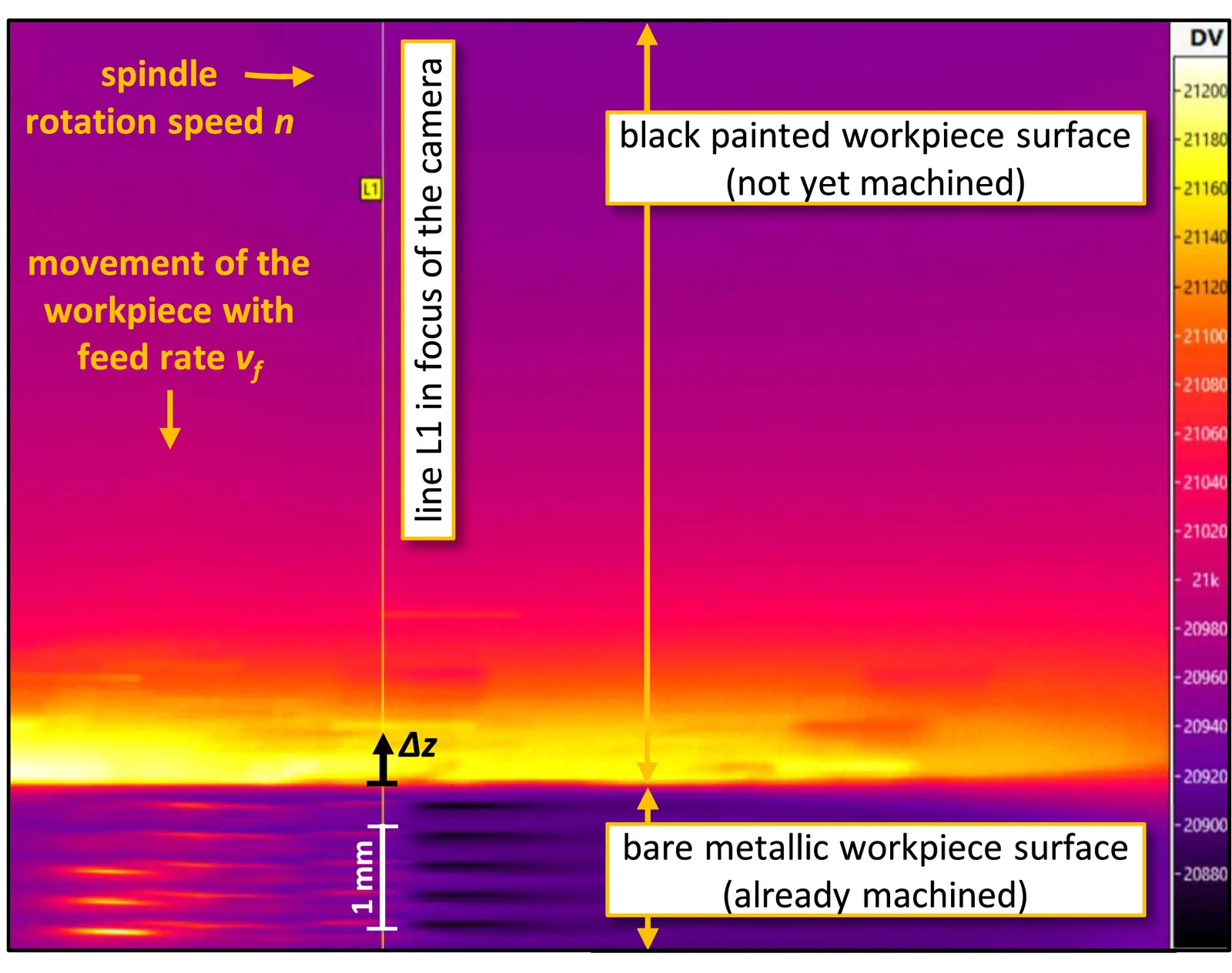
Exemplary thermographic image (machining test no 31 after cutting for 800 ms).

Temperature fields of the workpiece surface were measured by an infrared thermography camera, type ImageIR 8300 from InfraTec, with a microscope lens, type M = 1,0x WD 200, and the accompanying software-package IRBIS 3. A sample rate of 100 Hz and an integration time of 500 µs were set. Fig. 5 shows an exemplary thermographic image during the cutting process. The captured image displays 640 px (horizontal) x 512 px (vertical), with a scale of 17.11 µm/px determined using a prepared workpiece. The cutting tool is not visible in this close-up view, as the contact point with the workpiece is located much further to the left. The camera focus is set to the black painted workpiece surface and, due to the cylindrical shape of the workpiece, lies on the line L1 drawn in the image. Due to the shallow depth of field of infrared microscope lenses, a reliable measurement is only possible along this line. Furthermore, the image also shows that the machined bare metallic workpiece surface severely affects the signal due to a significantly lower emissivity.

**Fig. 6:**
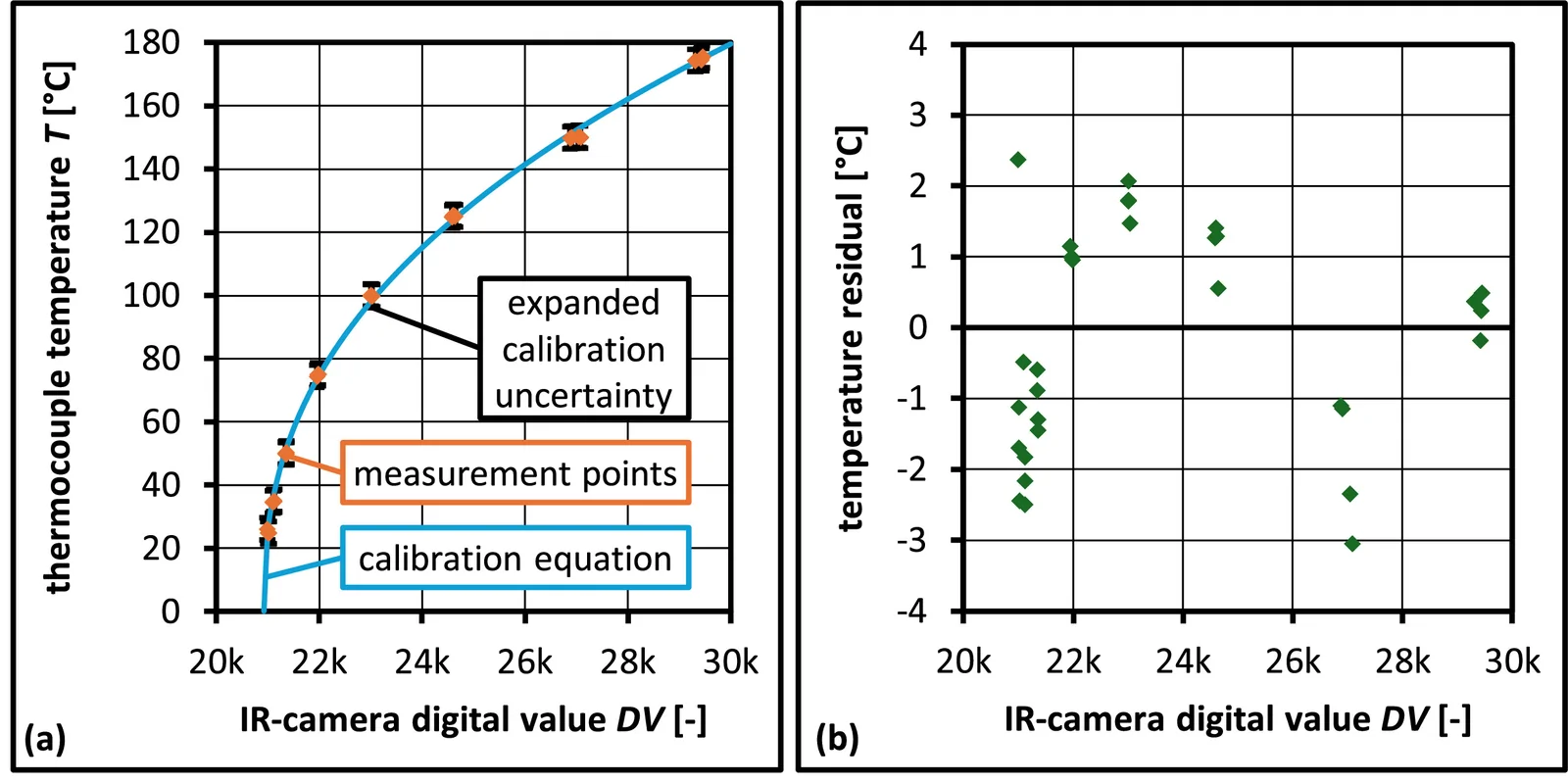
(a) calibration plot with non-linear fit and expanded calibration uncertainty,(b) corresponding temperature residuals distribution of the fit error.

To obtain temperature information from the digital value signal *DV*, a comparative calibration was performed. For this purpose, a workpiece was prepared in such a way that it could be heated to a defined temperature *T* on its black-painted surface within the field of view of the infrared camera, utilizing a resistance heating element and a PID temperature controller, type 600 from Gefran, with a connected type K thermocouple (“true” temperature). The temperature range from 25 °C to 175 °C was approached in eight steps (25; 35; 50; 75; 100; 125; 150; 175 °C) for both materials, with two repetitions each. Fig. 6a shows the resulting calibration plot and Eq. (1) provides the resulting calibration equation for both materials, which exhibits a coefficient of determination of *R²* = 0.999 for the investigated temperature range. The residual analysis is shown in Fig. 6b and the accuracy can be quantified by a Root Mean Square Error of RMSE = 1.54 °C, with a maximum absolute residual of 3.05 °C occurring at 150 °C. The error bars in Fig. 6a denote the expanded calibration uncertainty of ± 3.54 °C (k = 2, 95% confidence level), calculated via the geometric sum of the standard uncertainty of the reference thermocouple (± 1.5 °C / √3 ≈ 0.87 °C) and the regression error RMSE.

(1) $$T=4.2349{\left(DV-20922\right)}^{0.4112}$$

For data preparation, the digital values *DV* across the pixels along the line L1 were initially exported into Excel as data series for all recorded time steps and saved as raw data in .xlsx format. Time steps after the end of the cutting process, as well as the beginning of the measurement – where the workpiece had not yet moved far enough in the feed direction to produce an image analogous to Fig. 5 – were excluded. Additionally, there were time steps with justified outliers in the temperature values, which were also filtered out and will be discussed in more detail in the Limitations section. In a next step, the calibration equation was applied to subsequently calculate for each pixel the arithmetic mean of the temperature across all remaining time steps and the corresponding expanded uncertainty *U* for a two-sided 95% confidence interval according to ISO/IEC Guide 98–3:2008, type A (in Excel: *U* = STDEV.S(…) / sqrt (*sample size*) ∙ T.INV(0.975, *sample size* – 1)) as statistical repeatability to illustrate local temporal precision. Since the environmental conditions of the workshop were not climate-controlled, as is typical for the investigated turning process, the process-induced temperature rise *ΔT* was calculated by subtracting a reference temperature to obtain a quantity independent of the ambient conditions. This reference temperature was determined by calculating the mean value of the top 100 pixels of line L1 at the time step prior to cutting and is documented in the dataset. Across all machining tests, the mean reference temperature was 22.1 °C with a standard deviation of 3.5 °C. Using the determined scale, the pixels were ultimately expressed in a unit of length and shifted such that the distance to the newly generated surface begins at *Δz* = 0 mm, as indicated in Fig. 5. The temperature rise *ΔT* evaluated in this manner and its expanded uncertainty *U* over the distance to the machined surface *Δz* were saved together with the reference temperature as processed data in .xlsx format.

### 4.4 Experimental procedure

Prior to the start of the turning process, the machining parameters (spindle rotation speed *n*, feed rate *v_f_*, depth of cut *a_p_*) were set. The same set of machining parameters was deliberately applied to both workpiece materials to systematically isolate the influence of the thermophysical behavior under comparable material hardness conditions. While the selected depths of cut and feed rates largely overlap with standard specifications, intentionally extending into highly productive regimes, the cutting velocity range was chosen as a deliberate trade-off to approach high-speed aluminum specifications while simultaneously respecting the machinability of the steel alloy. After setting the machining parameters, the measurements on the measurement computer were started simultaneously with the initiation of the spindle movement via the machine tool control system. The cutting insert was replaced by a new one after each machining test in order to exclude tool wear effects.

## Limitations

**Fig. 7:**
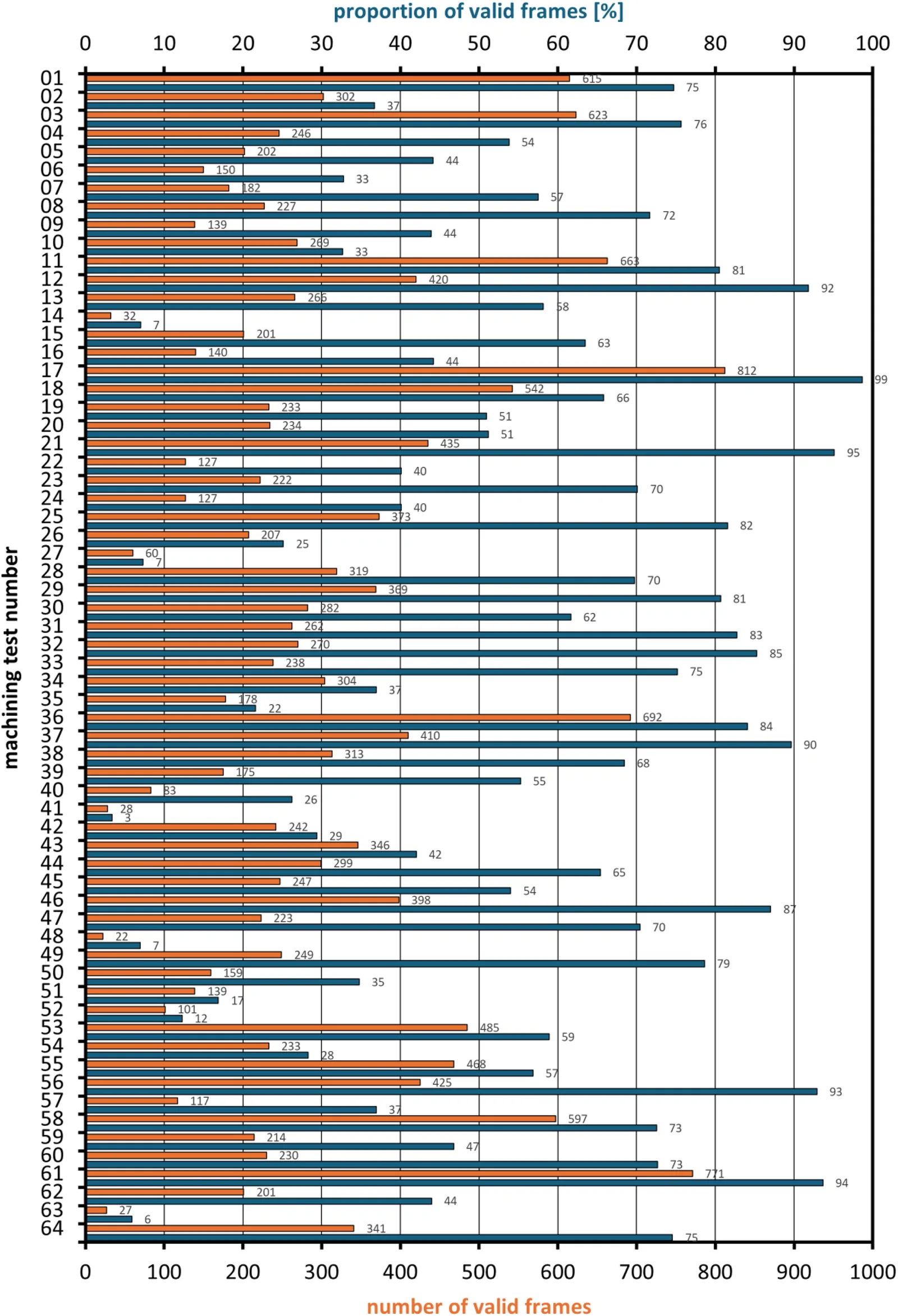
Number of valid frames and proportion relative to total frames during cutting.

Temperature measurement by means of infrared radiation heavily depends on keeping the measurement conditions identical during calibration and the cutting process. However, the chip formation process cannot be considered during calibration. From this, two limitations arose during creation of the dataset, both linked to the generated chips. Firstly, the occurrence of chips between the infrared camera and the measurement area on the workpiece could not fully be prevented. In cases where chips interfered with the measurement area, these thermographic images had to be removed from further analysis after being manually identified through visual inspection of each individual frame. Secondly, in rare cases, chips locally removed the black paint from the workpiece surface, and with this, the emissivity of these areas changed. These thermographic images were also manually identified and removed from further analysis. Thus, both limitations led to less thermographic images being analyzed. However, due to the high sample rate, for most of the conducted measurements, the amount of unaffected thermographic images still resulted in high quality data characterized by a low expanded Type A statistical repeatability error. Fig. 7 shows the number of remaining valid frames for each experiment (corresponding to the number of rows in the excel spreadsheet *raw_data*) along with the proportion of valid frames relative to the total number of captured frames during cutting. This total number depends on the feed rate and amounts to 317 frames (*v_f_* = 250 mm/min), 457 frames (*v_f_* = 450 mm/min), or 823 frames (*v_f_* = 650 mm/min). It is important to acknowledge that the manual frame selection introduces a degree of operator subjectivity, as no automated thresholding or independent verification was utilized. However, the visual artifacts causing exclusion (i.e., chip interference and localized paint removal within the evaluation area) were highly pronounced and unambiguous. This ensured a consistent exclusion process across the frame sequence.

## Ethics Statement

The authors declare that they have read and adhere to the ethical requirements for publication in Data in Brief. They also confirm that their work does not involve human subjects, animal experiments, or data collected from social media platforms.

## Credit Author Statement

**Lars Langenhorst:** Conceptualization, Methodology, Validation, Formal analysis, Investigation, Data Curation, Writing - Original Draft, Writing - Review & Editing, Funding acquisition. **Jens Sölter:** Conceptualization, Writing - Review & Editing, Supervision, Project administration, Funding acquisition
